# Supplementary material for: A Likelihood Approach for Real-Time Calibration of Stochastic Compartmental Epidemic Models
Source: PLoS Comput Biol. 2017 Jan 17;13(1):e1005257. doi: 10.1371/journal.pcbi.1005257 (PMC5240920; doi:10.1371/journal.pcbi.1005257)
Supplement: S1 File — (TAR.GZ) [file pcbi.1005257.s014.tar.gz › HSPH_Online-SI-Revision/EnKF07/n10-severe/n10-severe_PF_EnKF-summery.pdf]

peak −8	R0	$1/\theta_2$	inf	inflog	newly	newlylog	Reff	AttackRate
mean	65.5	66.6	9704.	1.3	8066.	1.2	58.6	145.6
median	63.7	55.9	3066.6	1.3	2682.5	1.2	55.7	152.1
Quantile .1	50.7	40.7	114.6	0.3	147.3	0.4	37.3	59.3
Quantile .9	77.8	110.8	23 461.4	2.3	12 482.1	1.9	78.1	190.6
36	40	9						
length	50							

peak −4	R0	$1/\theta_2$	inf	inflog	newly	newlylog	Reff	AttackRate
mean	67.8	55.3	374.3	0.6	229.2	0.6	65.2	180.7
median	67.	51.3	94.5	0.5	154.4	0.6	65.5	175.5
Quantile .1	53.8	29.7	67.1	0.3	69.4	0.4	53.4	133.9
Quantile .9	81.5	85.5	911.8	0.8	515.2	0.7	73.9	221.7
25	38	0						
length	50							

peak	R0	$1/\theta_2$	inf	inflog	newly	newlylog	Reff	AttackRate
mean	70.3	62.9	92.4	0.6	124.7	0.6	57.9	191.3
median	73.1	56.2	88.8	0.6	109.7	0.6	57.4	188.9
Quantile .1	53.4	30.	69.9	0.4	73.1	0.4	50.5	153.9
Quantile .9	85.9	97.6	116.	0.8	182.9	0.9	66.1	229.7
23	33	0						
length	50							

peak +4	R0	$1/\theta_2$	inf	inflog	newly	newlylog	Reff	AttackRate
mean	71.5	75.	176.5	0.4	227.5	0.5	57.	177.6
median	70.1	58.4	130.7	0.4	186.	0.5	56.4	177.6
Quantile .1	56.2	32.6	58.7	0.2	87.6	0.3	46.9	135.1
Quantile .9	87.3	148.9	370.9	0.6	407.4	0.7	68.	210.1
30	36	0						
length	50							

peak +8	R0	$1/\theta_2$	inf	inflog	newly	newlylog	Reff	AttackRate
mean	71.2	66.5	2756.	0.7	3567.2	0.8	56.2	173.5
median	66.2	59.4	472.1	0.6	954.9	0.7	56.1	169.2
Quantile .1	57.3	34.	114.6	0.3	376.9	0.4	42.5	131.
Quantile .9	90.	110.7	3751.5	1.2	4530.8	1.3	67.7	209.8
25	30	0						
length	50							
